# Prognostic Value of the PROFUND Index for 30-Day Mortality in Acute Heart Failure

**DOI:** 10.3390/medicina57111150

**Published:** 2021-10-23

**Authors:** Manuel Méndez-Bailón, Rosario Iguarán-Bermúdez, Lidia López-García, Beatriz Sánchez-Sauce, Pablo Pérez-Mateos, Julia Barrado-Cuchillo, Miguel Villar-Martínez, Santiago Fernández-Castelao, Jose Luis García-Klepzig, Manuel Enrique Fuentes-Ferrer, Alejandra García-García, Isidre Vilacosta, José María de Miguel-Yanes, José Manuel Casas-Rojo, Elpidio Calvo-Manuel, Emmanuel Andres, Noel Lorenzo-Villalba, on behalf of the Heart Failure and Pluripathology Groups from the Spanish National Society of Internal Medicine

**Affiliations:** 1 Servicios de Medicina Interna, Hospital Clínico San Carlos, Instituto de Investigación San Carlos (IdISSC), Universidad Complutense de Madrid, 28040 Madrid, Spain; manuelmenba@hotmail.com (M.M.-B.); draiguaran@gmail.com (R.I.-B.); pprezpablo@gmail.com (P.P.-M.); julia.barrado.c@gmail.com (J.B.-C.); donmiguelvillar@gmail.com (M.V.-M.); santiagofernandez91@gmail.com (S.F.-C.); jgcuenca85@gmail.com (J.L.G.-K.); elpidio.calvo@salud.madrid.org (E.C.-M.); 2 Servicio de Cardiología, Hospital Clínico San Carlos, 28040 Madrid, Spain; lidial02@ucm.es (L.L.-G.); i.vilacosta@gmail.com (I.V.); 3 Servicio de Medicina Interna, Fundación Hospital Alcorcón Alcorcón, 28922 Madrid, Spain; beatrizsauce@gmail.com; 4 Servicio de Medicina Preventiva, Instituto de Investigación San Carlos (IdISSC), Hospital Clínico San Carlos, 28040 Madrid, Spain; mfuentesferrer@gmail.com; 5 Servicio de Medicina Interna, Hospital Universitario Gregorio Marañón, 28007 Madrid, Spain; alejandra.garcia.garcia@salud.madrid.org (A.G.-G.); josemaria.demiguel@salud.madrid.org (J.M.d.M.-Y.); 6 Servicio de Medicina Interna, Hospital Infanta Cristina, 28981 Madrid, Spain; jm.casas@gmail.com; 7 Service de Médecine Interne, Diabète et Maladies Métaboliques, Hôpitaux Universitaires de Strasbourg, 67091 Strasbourg, France; emmanuel.andres@chru-strasbourg.fr

**Keywords:** heart failure, PROFUND index, pluripathology

## Abstract

*Background and Objectives:* The prevalence and incidence of heart failure (HF) have been increasing in recent years as the population ages. These patients show a distinct profile of comorbidity, which makes their care more complex. In recent years, the PROFUND index, a specific tool for estimating the mortality rate at one year in pluripathology patients, has been developed. The aim of this study was to evaluate the prognostic value of the PROFUND index and of in-hospital and 30-day mortality after discharge of patients admitted for acute heart failure (AHF). *Materials and Methods*: A prospective multicenter longitudinal study was performed that included patients admitted with AHF and ≥2 comorbid conditions. Clinical, analytical, and prognostic variables were collected. The PROFUND index was collected in all patients and rates of in-hospital and 30-day mortality after discharge were analyzed. A bivariate analysis was performed with quantitative variables between patients who died and those who survived at the 30-day follow-up. A logistic regression analysis was performed with the variables that obtained statistical significance in the bivariate analysis between deceased and surviving subjects. *Results:* A total of 128 patients were included. Mean age was 80.5 +/− 9.98 years, and women represented 51.6%. The mean PROFUND index was 5.26 +/− 4.5. The mortality rate was 8.6% in-hospital and 20.3% at 30 days. Preserved left ventricular ejection fraction was found in 60.9%. In the sample studied, there were patients with a PROFUND score < 7 predominated (89 patients (70%) versus 39 patients (31%) with a PROFUND score ≥ 7). Thirteen patients (15%) with a PROFUND score < 7 died versus the 13 (33%) with a PROFUND score ≥ 7, *p* = 0.03. Twelve patients (15%) with a PROFUND score < 7 required readmission versus 12 patients (35%) with a PROFUND score ≥ 7, *p* = 0.02. The ROC curve of the PROFUND index for in-hospital mortality and 30-day follow-up in patients with AHF showed AUC 0.63, CI: 95% (0.508–0.764), *p* <0.033. *Conclusions:* The PROFUND index is a clinical tool that may be useful for predicting short-term mortality in elderly patients with AHF. Further studies with larger simple sizes are required to validate these results.

## 1. Introduction

The prevalence and incidence of heart failure (HF) have been increasing in recent years as the population ages. The prevalence of HF is between 2 and 3%, reaching 10–20% in patients between 70 and 80 years of age [1,2,3]. This high prevalence creates an increase in the demand for health care, making HF the most important cause of admission to Spanish hospitals and representing 5% of all hospitalizations [2,3]. The frequency of hospital readmission for this disease ranges from 30 to 60% in the first six months following hospital discharge [4]. These patients, who are often suffering from multiple pathologies, require more intense care. The comorbid conditions that are significantly increased include chronic renal failure, severe hematological disorders, malnutrition, psychiatric disorders, and pressure ulcers. These comorbidities could affect treatment and have an important prognostic impact, leading to more hospital admissions, worsening quality of life, and increased mortality [5,6].

It is in this context that the concept of pluripathology arises, identifying a population of patients with two or more chronic diseases with an equivalent degree of complexity. Pluripathology patients constitute a heterogeneous group of patients with a series of easily identifiable common characteristics: greater complexity, clinical vulnerability, frailty, mortality, functional deterioration, polypharmacy, poorer health-related quality of life, and dependency [7,8].

The prevalence of pluripathology patients in hospital setting varies between 25–50%. HF is the most frequent defining category of pluripathology patients with a prevalence of 72–77%. In addition, there is a high prevalence among pluripathology patients of other cardiovascular comorbidities related to HF (ranging from 30% to 70%) [9]: arterial hypertension, diabetes, dyslipidemia, and atrial fibrillation.

In recent years, the PROFUND index (Supplementary Table S1), a specific tool for estimating the mortality risk at one year in pluripathology patients, has been developed. This index was developed and validated in a hospital-based multicenter cohort recruited in 36 Spanish hospitals. A total of 1632 patients were included and followed for one year, and the index was subsequently derived and validated. This scale stratifies 12-month mortality and is based on nine clinical, analytical, and socio-familial dimensions [8,9]. The index stratifies pluripathology patients into four risk groups according to the scores obtained, with mortality ranging from 12–14% in the lowest risk stratum to 61–68% in patients with 11 or more points [8,9]. However, this scale has not been evaluated in pluripathology patients with acute HF.

The aim of this study was to evaluate the value of the PROFUND index in predicting in-hospital and 30-day mortality after the discharge of patients admitted for acute heart failure. 

## 2. Methodology

### 2.1. Design, Study Population, Variables, Data Collection and Analysis, and Ethical Aspects of the Study

#### 2.1.1. Design and Study Population

This was a nationwide prospective multicenter cohort study from the HF study group and the Pluripathologic Patients and Advanced Age group of the Spanish Society of Internal Medicine. All patients who were >18 years old and who had been admitted for HF as the main diagnosis with two or more comorbid conditions admitted in internal medicine services were consecutively included from September 2020 to May 2021. Inclusion criteria included patients admitted with a principal diagnosis of HF, two or more comorbid conditions, and NT-pro-brain natriuretic peptide (NT-proBNP) > 300 pg/mL on admission and/or on arrival at the emergency department. Absence of informed consent from either the patient or legal representative was considered an exclusion criteria.

#### 2.1.2. Data Collection

Data from patients admitted for acute heart failure (AHF) to the internal medical department were prospectively collected by researchers (medical and nursing personnel) during the months of September 2020 to May 2021. The following parameters were collected: date of birth, sex, toxic habits, arterial hypertension, dyslipidemia, diabetes mellitus, left ventricular ejection fraction (LVEF), New York Heart Association (NYHA) dyspnea functional class at baseline (two weeks prior to admission), presence of pluripathology (defined by the presence of two or more chronic diseases of the clinical categories listed in Supplementary Table S2), NT-proBNP levels, and chest X-ray. Data regarding the patient’s HF history, such as date of admission, date of readmission for HF, date of death, mortality due to cardiovascular and non-cardiovascular causes during follow-up, disposition, and treatment at discharge, were collected.

In addition, data about all-cause mortality and readmissions at 30 days as well as PROFUND index scores were collected (Table 1). The PROFUND index is calculated from the score obtained from the values for each of the variables included in Table 1. This index scores from 0 to 30 points as maximum and classifies multipathologic patients from the prognostic point of view of survival at one year (Table 1). Subjects with scores equal to or greater than 7 points are those with the highest risk of mortality equal to or greater than 45% during the first year of follow-up.

#### 2.1.3. Statistical Analysis

The clinical characteristics of the sample including qualitative and quantitative variables were evaluated through a descriptive analysis. A bivariate analysis was performed with variables between patients who died and survived at 30-day follow-up. We also conducted a bivariate analysis between patients with PROFUND index scores ≥7 and ˂7 points, as this is the cutoff defining patients who are at a higher mortality risk. The chi-square test was used for qualitative variables, and Student’s *t* test was used for quantitative variables.

A receiver operating curve (ROC) curve analysis was performed to evaluate the area under the curve for 30-day mortality of the PROFUND index and NT-proBNP values. The cutoff point was calculated for both curves by considering the highest sensitivity and specificity values. Afterwards, a logistic regression analysis was performed with the variables of PROFUND index points and NT-proBNP. We performed a binary regression logistic analysis taking into account the sample characteristics with NT-proBNP and PROFUND index values using the Hosmer–Lemeshow test. The analysis was performed using SPSS program 26.0.

#### 2.1.4. Ethical Aspects

The project was submitted to the Clinical Research Ethics Committee of each hospital. All investigators and personnel involved in the project were aware of and adhered to local and international regulations in the field of ethical considerations for human experimentation, including the Helsinki declaration with its revisions, the Belmont report, and other related documents.

Data confidentiality was maintained in accordance with the Data Protection Law (Organic Law 5/92 of 29 October on the regulation of the automated processing of personal data, BOE 30 October 1992, modified by Organic Law 15/1999, of 13 December, on the Protection of Personal Data and Law 41/2002, of 14 November).

All patients, in addition to being informed verbally in detail about the project, provided their informed consent in writing before being included in the study. This study was approved by the Ethical Committee from Fundanción Hospital Alcorcon Madrid on 11 June 2019.

## 3. Results

A total of 128 patients with a main diagnosis of AHF were included. The mean age was 80.5 +/− 9.98 years, and women represented 51.6% of the sample. The mean PROFUND index was 5.26 +/− 4.5. The in-hospital mortality rate was 8.6% and 20.3% at 30 days post-discharge. In the population studied, 60.9% patients presented a preserved LVEF, 21.1% presented reduced LVEF, and 18% presented slightly reduced LVEF. The median NT-proBNP in the ED was 5815 pg/mL. The most frequent HF etiology was hypertension, and the most frequent associated comorbid conditions were hypertension (90%), dyslipidemia (76%), and atrial fibrillation (65%). A total of 43.7% of patients had been admitted two or more times in the last year for acute heart failure (Table 2).

Of the patients included in the registry, 23 (18%) had a previous SARS-COV2 infection; 26 patients (20%) died during admission or at the 30-day follow-up. In the first 30 days, 24 patients (21%) were readmitted, of whom 14 (58.3%) were admitted for cardiac decompensation. 

In the sample studied, patients with a PROFUND score of < 7 predominated, as this accounted for 89 patients (70%) versus the 39 patients (31%) with a PROFUND score ≥ 7. In the first group 13 patients (15%) died versus 13 (33%) in the second group, *p* = 0.03. In relation to readmission, 12 patients (15%) with PROFUND scores < 7 were readmitted versus 12 patients (35%) with PROFUND scores ≥ 7, *p* = 0.021. 

**Figure 1:**
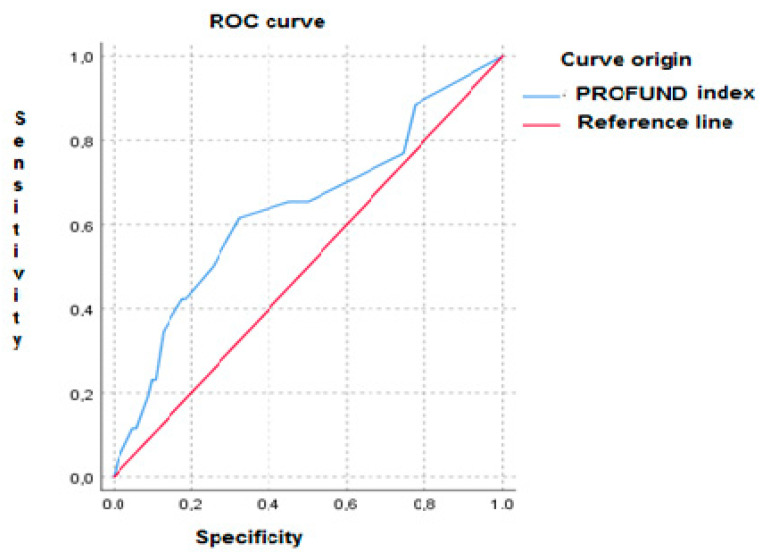
ROC curve of the PROFUND index for in-hospital mortality and 30-day follow-up in patients with AHF.

Figure 1 shows the ROC curve of the PROFUND index for in-hospital mortality and 30-day follow-up in patients with acute heart failure (AUC 0.63, CI: 95% (0.508–0.764), *p* < 0.033).

**Figure 2:**
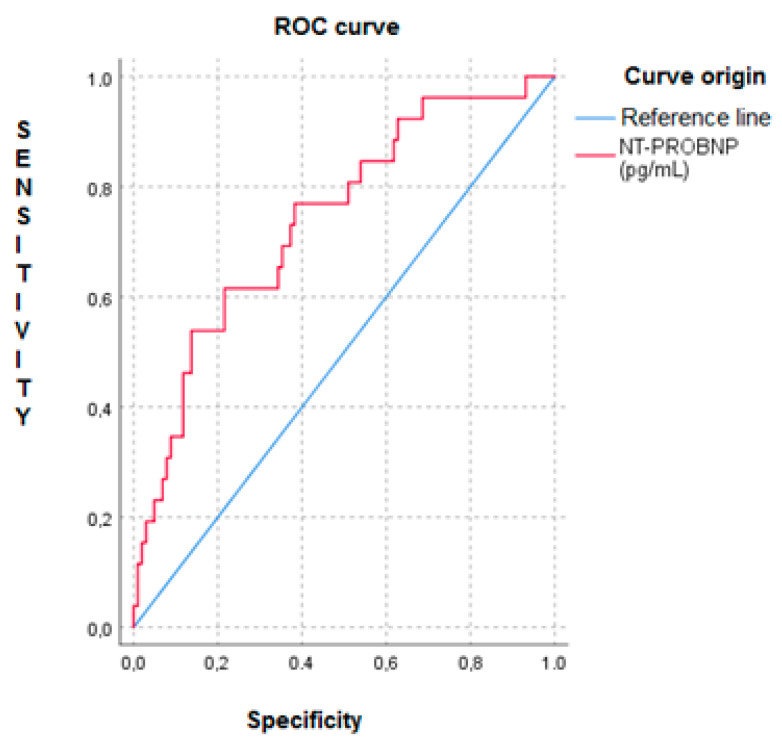
ROC curve of the NT-proBNP levels for in-hospital mortality and 30-day follow-up in patients with AHF.

Figure 2 shows the ROC curve of the NT-proBNP levels for in-hospital mortality and 30-day follow-up in patients with AHF (AUC 0.73, 95% CI (0.63–0.847), *p* < 0.001).

In the comparative analysis between patients with mortality and survival at the 30-day follow-up, it was observed that PROFUND index, NT-proBNP, creatinine, and sodium values were the variables associated with mortality (Table 3).

PROFUND index and NT-proBNP were included in the logistic regression analysis, as shown in Table 4.

## 4. Discussion

This study suggests that the PROFUND index could be a helpful prognostic tool to assess the risk of 30-day mortality in patients with HF. In this sense, this study is the first to evaluate the prognostic value of this index in patients with AHF and several associated chronic diseases in the first 30 days of follow up [8,9,10]. In the population that was studied, the main comorbidities were hypertension, dyslipidemia, atrial fibrillation, diabetes, and anemia, as reported by other authors [7]. These comorbidities have already been associated with poor prognosis in patients with heart failure [8,9,10].

The PROFUND index had previously been evaluated by other authors and has been shown to be a good tool for determining the annual prognosis of chronic multipathologic patients [8,9,10]. However, this index had not been evaluated in patients with AHF. The 30-day prognostic assessment is also novel since it has usually been validated at the one-year follow-up [10]. Another study reported the accuracy of PROFUND index in predicting the mortality of polypathological patients during a 4-year follow up period [11,12,13,14,15]. All of these factors make the PROFUND index an attractive clinical tool because it offers the possibility of a rapid, comprehensive, multidimensional assessment of an elderly HF patient. Comprehensive assessment has been a recommendation stated in many consensus reports on the care of elderly HF patients, and the PROFUND index may be a useful tool. [4].

In our results, the AUC ROC curve of the PROFUND index for 30-day mortality was statistically significant but had a modest area under the curve. These findings could be due to a self-limitation of the PROFUND index s in predicting short-term mortality in patients with HF. Results provided by other authors such as Bernabeu-Wittel et al. [8] show that at one year of follow-up in 1600 patients, the area under the ROC curve obtained was 0.77 compared to the value of 0.63 obtained in our series. Perhaps the smaller sample size used in our study and the shorter follow-up period may explain the differences obtained between the aforementioned studies.

In light of our results, NT-proBNP levels are an independent short-term predictor of mortality in patients with HF and associated comorbid conditions. This finding, even if well known, has not been analyzed in patients with HF and pluripathologic conditions. Approximately 50% of our patients presented with chronic renal failure, which could have caused overestimates in the NT-proBNP levels. In this sense, the NT-proBNP values and the PROFUND index could better predict short-term mortality in these patients.

The high mortality rate of the patients included in our series is noteworthy. Approximately 20% of the patients died in the first 30 days. This study was conducted during the COVID-19 pandemic, which may have influenced our results. The quarantine prompted by the pandemic to attempt to prevent the infection of this group probably affected the functionality of the patients. The difficulty in the ambulatory control of this disease with virtual management in some of these patients could have influenced the high rate of events observed in the series. However, we do not know whether this hypothesis is true since we have not observed a worse clinical course in patients with HF and previous COVID-19 infection.

A limitation of our study is the small sample size of our series to evaluate mortality. Further studies should include more patients to increase the statistical power. We also did not compare the prognostic value of the PROFUND index with other validated scales in AHF (such as the MEESSI) or in HF (such as SEATTLE and MAGGIC). We do not know how the outpatient follow-up of patients was performed, and this could have influenced their prognosis.

## 5. Conclusions

In conclusion, the PROFUND index is a clinical tool that may be useful for predicting short-term mortality in elderly patients with AHF. Further studies are needed to confirm these findings with larger sample sizes.

## Figures and Tables

**Table 1:** PROFUND scale variables.

PROFUND Index	
VARIABLE	Points
Age ≥ 85 years	3
Clinical features - Active neoplasia; - Dementia; - NYHA functional class III-IV or mMRC 3–4; - Delirium during the last hospital admission.	6 3 3 3
Hb < 10 g/dL	3
Socio-familial features - Barthel index < 60; - Lack of caregiver or caregiver other than partner.	4 2

**Table 2:** General characteristics of patients included in the PROFUND-IC registry.

Variable	
Age	80.5 +/− 9.98 years
Sex (female)	66 (52%)
**Comorbid Conditions**	
Hypertension	114 (90%)
Dyslipidemia	97 (76%)
Atrial fibrillation	82 (65%)
Chronic renal failure	60 (47%)
Diabetes	59 (46%)
Ischemic heart disease	45 (43%)
Anemia (Hb < 10 g/dL)	45 (35%)
COPD	26 (20%)
Sleep apnea	18 (14%)
Solid tumors	9 (7%)
Cognitive impairment	14 (11%)
**Heart Failure Etiology**	
Hypertensive	53 (41%)
Ischemic	40 (31%)
Valvular	16 (12%)
Amyloidosis	6 (5%)
Other	13 (10%)
**NYHA Functional Class**	
Class I	5 (4%)
Class II	80 (62%)
Class III	42 (33%)
Class IV	1 (0.8%)
**Left ventricular ejection fraction**	
40–49%	23 (18%)
≥50%	78 (61%)
<40%	27 (11%)
Functional status (Barthel)	71.4+/− 27.83
**Laboratory Results upon Admission**	
Hemoglobin	11.8 +/− 1.9 g/dL
Serum creatinine	1.4 +/− 0.7 mg/dL
Sodium	139.6 +/− 4.7 mmol/L
Potassium	4.3 +/− 0.6 mmol/L
NT-proBNP	5815.5 pg/mL *
**Treatment upon Admission**	
Furosemide	104 (85%)
Beta blockers	74 (61%)
Angiotensin converting enzyme inhibitors	35 (29%)
Mineralocorticoids	34 (28%)
Angiotensin II receptor antagonist	21 (17%)
Sacubutril-Valsartan	8(6.6%)
SGTL-2 inhibitors	8 (6.6%)
Cardiovascular cause of death	8 (2%)

Legend: COPD: chronic obstructive pulmonary disease; Hb: hemoglobin; NT-proBNP: N-terminal pro-B natriuretic peptide; SGTL-2: sodium-glucose cotransporter-2. * NT-proBNP expressed as median.

**Table 3:** Comparative analysis of mortality and survival at 30 days of follow up.

Variable	Expired (N = 26)	Survived (N = 102)	*p*
Age	83.2 +/− 9.8 years	79.8 +/− 9.9 years	0.0119
Sex (Males)	21%	79%	0.516
Left ventricular ejection fraction	46.6 +/− 13.1%	48.2 +/− 11.3%	0.561
Hemoglobin	11.9 +/− 2.0 g/dL	11.8 +/− 1.9 g/dL	0.782
Creatinine	1.8 +/− 1.1 mg/dL	1.3 +/− 0.6 mg/dL	0.001
NT-proBNP	184500 +/− 3790 pg/mL	7953 +/− 965 pg/mL	<0.001
Sodium	136.4 +/− 4.8 mmol/L	140.4 +/− 4.3 mmol/L	<0.001
PROFUND index	7.2 +/− 5.1	4.7 +/− 4.2	0.001

Legend: NT-proBNP: N-terminal pro-B natriuretic peptide.

**Table 4:** Logistic regression for 30-day mortality factors in patients included in the PROFUND-IC registry.

Variable	OR	CI95%	*p*
PROFUND index ≥ 9 points	2.39	0.87–6.51	0.088
NT-proBNP ≥ 14,000 pg/mL	4.31	1.59–11.67	0.004

Legend: NT-proBNP: N-terminal pro-B natriuretic peptide.

## Supplementary Materials

The following are available online at https://www.mdpi.com/article/10.3390/medicina57111150/s1, Table S1: Mortality according to PROFUND index score, Table S2: Clinical categories for the identification of pluripathological patients.

## References

[1] Cortina A., Reguero J., Segovia E., Lambert J.L., Cortina R., Arias J.C., Vara J., Torre F. (2001). Prevalence of heart failure in Asturias (a region in the North of Spain). Am. J. Cardiol..

[2] Banegas J.R., Rodríguez-Artalejo F., Guallar-Castillón P. (2006). Situación epidemiológica de la insuficiencia cardiaca en España. Rev. Esp. Cardiol. Supl..

[3] Fernández Gassó M.L., Hernando-Arizaleta L., Palomar-Rodríguez J.A., Soria-Arcos F., Pascual-Figal D.A. (2017). Trends and characteristics of hospitalization for heart failure in a population setting from 2003 to 2013. Rev. Esp. Cardiol..

[4] Dharmarajan K. (2016). Comprehensive strategies to reduce readmissions in older patients with cardiovascular disease. Can. J. Cardiol..

[5] Conde-Martel A., Hernández-Meneses M. (2016). Prevalencia y significado pronóstico de la comorbilidad en la insuficiencia cardiaca. Rev. Clin. Esp..

[6] Formiga F., Chivite D., Conde A., Ruiz-Laiglesia F., Franco Á.G., Bocanegra C.P., Manzano L., Pérez-Barquero M.M., RICA Investigators (2014). Basal functional status predicts three-month mortality after a heart failure hospitalization in elderly patients—The prospective RICA study. Int. J. Cardiol..

[7] García-Morillo J.S., Bernabeu-Wittel M., Ollero-Baturone M., Aguilar-Guisad M., Ramírez-Duque N., de la Puente M.A., Limpo P., Romero-Carmona S., Cuello-Contreras J.A. (2005). Incidencia y características clínicas de los pacientes con pluripatología ingresados en una unidad de medicina interna. Med. Clin..

[8] Bernabeu-Wittel M., Ollero-Baturone M., Moreno-Gaviño L., Barón-Franco B., Fuertes A., Murcia-Zaragoza J., Ramos-Cantos C., Alemán A., Fernández-Moyano A. (2011). Development of a new predictive model for polypathological patients. The PROFUND index. Eur. J. Intern. Med..

[9] Bernabeu-Wittel M., Barón-Franco B., Nieto-Martín D., Moreno-Gaviño L., Ramírez-Duque N., Ollero-Baturone M. (2017). Estratificación pronóstica y abordaje asistencial de los pacientes pluripatológicos. Rev. Clin. Esp..

[10] Moretti D., Buncuga M.G., Laudanno C.D., Quiñones N.D., Scolari Pasinato C.M., Rossi F.E. (2021). Índice PROFUND y mortalidad intrahospitalaria en pacientes pluripatológicos. Análisis post-hoc [PROFUND index and intrahospital mortality in plurypathological patients. A post-hoc analysis]. Medicina.

[11] Bernabeu-Wittel M., Moreno-Gaviño L., Ollero-Baturone M., Barón-Franco B., Díez-Manglano J., Rivas-Cobas C., Murcia-Zaragoza J., Ramos-Cantos C., Fernández-Moyano A. (2016). Validation of PROFUND prognostic index over a four-year follow-up period. Eur. J. Intern. Med..

[12] Moretti D., Buncuga M.G., Laudanno C.D., Quiñones N.D., Scolari Pasinato C.M., Rossi F.E. (2020). Índice PROFUND y valoración global subjetiva. Valor pronóstico en pacientes pluripatológicos internados. Medicina.

[13] López-Garrido M.A., Martín-Portugués I.A., Becerra-Muñoz V.M., Orellana-Figueroa H.N., Sánchez-Lora F.J., Morcillo-Hidalgo L., Jiménez-Navarro M.F., Gómez-Doblas J.J., de Teresa-Galván E., García-Pinilla J.M. (2017). Prevalencia de pluripatología y valor pronóstico del índice PROFUND en una unidad de hospitalización de Cardiología. Rev. Clin. Esp..

[14] Colombo P.B., Nieto Martín M.D., Pascual de la Pisa B., José García Lozano M., Ángeles Ortiz Camúñez M., Wittel M.B. (2014). Validación de un modelo pronóstico para pacientes pluripatológicos en atención primaria: Estudio PROFUND en atención primaria. Aten Primaria..

[15] Ruiz-Laiglesia F.J., Sánchez-Marteles M., Pérez-Calvo J.I., Formiga F., Bartolomé-Satué J.A., Armengou-Arxé A., Lopez-Quiros R., Perez-Silvestre J., Serrado-Iglesias A., Montero-Pérez-Barquero M. (2014). Comorbidity in heart failure. Results of the Spanish RICA Registry. QJM.

