# Supplementary material for: Prognostic Value of the PROFUND Index for 30-Day Mortality in Acute Heart Failure
Source: Medicina (Kaunas). 2021 Oct 23;57(11):1150. doi: 10.3390/medicina57111150 (PMC8618627; doi:10.3390/medicina57111150)
Supplement: Supplementary file 1 [file medicina-57-01150-s001.zip › medicina-1385360-supplementary.pdf]

Supplementary material

Table S1. Mortality according to PROFUND index score.

**PROFUND (Points)**	**Mortality at One Year (%)**
0-2	12,0-14,6
3-6	21,5-31,5
7-10	45-50
≥11	61,3-68

Table S2. Clinical categories for the identification of pluripathological patients.

**Clinical Categories for the Identification of Pluripathological Patients**
CATEGORY A
A1. Heart failure that in a clinically stable situation has been in NYHA II (symptoms with usual physical activity)<br>A2. Ischemic cardiomyopathy
CATEGORY B
B.1. Vasculitis and systemic autoimmune diseases<br>B.2. Chronic kidney disease defined by glomerular filtration rate < 60ml/m or proteinuria persisting for three months
CATEGORY C
C.1. Chronic respiratory disease that in a situation of clinical stability presented with grade II dyspnea mMRC3 (dyspnea at usual pace in flat), FEV1
CATEGORY D
D.1. Chronic inflammatory bowel disease<br>D.2. Chronic liver disease with signs of liver failure or portal hypertension
CATEGORY E
E.1. Stroke<br>E.2. Neurological disease with permanent motor deficit limiting basic daily living activities (Barthel index < 60)<br>E.3. Neurological disease with permanent cognitive impairment, at least moderate (Pfeiffer ≥ 5 or more errors)
CATEGORY F
F.1. Symptomatic peripheral arterial disease<br>F.2. Diabetes mellitus with proliferative retinopathy or symptomatic neuropathy
CATEGORY G
G.1. Chronic anemia due to digestive losses or acquired hematologic disease<br>G.2. Active solid or hematologic neoplasm that does not require treatment with curative intent
CATEGORY H
H.1. Chronic osteoarticular disease that causes by itself a limitation for basic daily living activities (Barthel index < 60)

FEV1: force expiratory volume.
